# Genetic and phenotypic characterization of the plasmid-encoded NDM-80 metallo-β-lactamase in *Escherichia coli* isolated from a pediatric patient

**DOI:** 10.3389/fmicb.2026.1770791

**Published:** 2026-03-02

**Authors:** Jinlan Zhou, Qing Meng, Qimeng Fan, Weiwei Yang, Li Ding, Yan Guo, Fupin Hu, Guoping Lu, Gangfeng Yan

**Affiliations:** 1 Department of Critical Care Medicine, Shanghai Institute of Infectious Disease and Biosecurity, Children’s Hospital of Fudan University, Fudan University, Shanghai, China; 2 National Children’s Medical Center, Fudan University, Shanghai, China; 3 National Health Commission Key Laboratory of Neonatal Diseases, Fudan University, Shanghai, China; 4 Department of Clinical Microbiology Laboratory, Shenzhen Children’s Hospital, Shenzhen, Guangdong, China; 5 Department of Pediatric Intensive Care Unit, Shenzhen Children’s Hospital, Shenzhen, Guangdong, China; 6 Institute of Antibiotics, Huashan Hospital, Fudan University, Shanghai, China; 7 Key Laboratory of Clinical Pharmacology of Antibiotics, Ministry of Health, Shanghai, China; 8 Joint Laboratory of Hospital & Enterprise for Pathogen Diagnosis of Drug-Resistant Bacterial Infections and Innovative Drug R&D, Shanghai, China

**Keywords:** *bla*_*NDM–80*_, carbapenem-resistant, *E. coli*, pediatric, ST155

## Abstract

**Introduction:**

Carbapenem-resistant Enterobacterales (CRE) strains carrying *bla*_*NDM*_ variants pose a significant threat to the health of infected patients worldwide.

**Methods:**

This study isolated a carbapenem-resistant *Escherichia coli* (*E. coli*) strain carrying *bla*_*NDM–*80_ from a patient in an intensive care unit in China. Antimicrobial susceptibility testing, whole-genome sequencing (WGS), plasmid transformation assay, cloning experiment, and steady-state kinetic determinations were performed to investigate antimicrobial susceptibility, the characteristics of the genetic environment, the mechanism of resistance gene transmission, resistance gene function, and antibiotic hydrolysis ability.

**Results:**

The results indicated that *E. coli* carrying *bla*_*NDM–*80_ showed significant resistance to all β-lactam antibiotics except for aztreonam, aztreonam-avibactam, and cefiderocol. WGS analysis revealed that the strain belonged to sequence type (ST) 155 and the O4:H51 serotype. The *bla*_*NDM–*80_ was carried by an unconjugated plasmid, and its complete genetic structure was found to be *IS*5-*bla*_*NDM–*80_-*ble*-*trpF*-*dsbD*-*IS*26-*nmuD*-*IS*Kox3. The *bla*_*NDM–*80_ had single amino acid substitutions such as V88L, D130N, and M154L compared to *bla*_*NDM–*1_, whereas it only had the D130N mutation compared to *bla*_*NDM–*5_. The *bla*_*NDM–*80_ and *bla*_*NDM–*5_ had similar antimicrobial resistance profiles. However, in the absence of the native promoter, the minimum inhibitory concentrations (MICs) of *bla*_*NDM–*80_ for imipenem, meropenem, cefepime, and cefiderocol were twice as high as those of *bla*_*NDM–*1_. Steady-state kinetic determinations revealed that NDM-80 likely had higher hydrolytic activity against imipenem, meropenem, cefepime, and cefiderocol than NDM-1.

**Discussion:**

This study is the first to report on the emergence of the *bla*_*NDM–*80_ variant, shedding light on its functional mechanism. Our findings enrich the repertoire of NDM resistance genes and highlight the need for increased surveillance of pathogens harboring *bla*_*NDM*_ variants.

## 1 Introduction

Carbapenem-resistant Enterobacterales (CRE) have emerged and spread rapidly worldwide, posing a significant threat to human health (Yigit et al., 2001; Mackow and van Duin, 2024). The most common clinical isolates of CRE are *Klebsiella pneumoniae* and *Escherichia coli* (Zhang et al., 2018). In 2024, the World Health Organization listed CRE as a critical priority pathogen for the urgent development of new antibiotics (World Health Organization [WHO], 2024). Epidemiological research has shown that the overall annual incidence of CRE in the United States is 2.93 per 100,000 people, whereas in China it is 4 per 10,000 hospital discharges (Guh et al., 2015; Zhang et al., 2018). Furthermore, clinical studies indicate that, compared with infections caused by carbapenem-sensitive Enterobacterales, those caused by CRE are associated with a higher mortality rate (Stewardson et al., 2019).

The main mechanisms causing CRE resistance are porin mutations, efflux pump overexpression, and carbapenemase production. Of these, carbapenemase production is the most important CRE resistance mechanism, and it is divided into classes A, B, and D according to the Ambler classification system. Classes A and D consist of serine β-lactamases, while class B comprises metallo-β-lactamases (MBLs), such as New Delhi metallo-β-lactamase (Al-Marzooq et al., 2024; Dixon et al., 2022). *K. pneumoniae* isolates from adults primarily produce class A enzymes, predominantly KPC. KPC-2 is the most prevalent in China, whereas KPC-3 and KPC-2 predominate jointly in the Americas and Europe (Falagas et al., 2025). In pediatric patients, metallo-β-lactamases (including NDM-1 and NDM-5) are the primary resistance mechanism, followed by KPC and class D carbapenemases (with OXA-232 and OXA-48 being the most common) (Zhou et al., 2024). The primary resistance mechanism identified in *E. coli* isolates from both adult and pediatric patients was MBL production, with NDM-5 and NDM-1 being the most commonly detected (Xiong et al., 2023; Boutzoukas et al., 2023). MBLs can hydrolyze almost all β-lactam antibiotics except aztreonam, and new β-lactamase inhibitors are ineffective against them, posing a significant challenge for clinical treatment (Theuretzbacher et al., 2021; Feng et al., 2017). NDM is a typical representative of MBLs. Since NDM-1 was first isolated from *K. pneumoniae* in 2008 (Yong et al., 2009), more than 90 NDM variants have been identified worldwide, according to the NCBI database (National Center for Biotechnology Information [NCBI], 2025). These variants exhibit different levels of hydrolytic activity against β-lactam antibiotics. For instance, NDM-5 exhibits increased hydrolytic activity against carbapenem antibiotics compared to NDM-1 (Hornsey et al., 2011), whereas NDM-9 and NDM-35 demonstrate enhanced hydrolytic activity against cefiderocol (Poirel et al., 2022; Gaillot et al., 2023). Therefore, strengthening surveillance of NDM-carrying pathogens is essential to track their resistance profiles and guide clinical therapy.

This study describes the recently identified *bla*_*NDM*_ variant (*bla*_*NDM–*80_) carried by *E. coli* and isolated from a fecal specimen taken from a 1-year-old patient in the pediatric intensive care unit in China. Through antimicrobial susceptibility testing, whole-genome sequencing, molecular cloning, and enzymatic kinetics assays, we explored its genetic characteristics and resistance mechanisms.

## 2 Materials and methods

### 2.1 Bacterial strain

A strain of *E. coli* carrying a new NDM variant (designated *E. coli* SE-eco-21) was isolated from a 1-year-old boy hospitalized with severe cough at the pediatric intensive care unit of the Children’s Hospital of Shenzhen. The strain was obtained from stool after 5 days hospitalization and no pathogen was isolated from the sputum. The patient was only successively treated with the ceftazidime and ceftriaxone during the hospitalization. Interestingly, on the seventh day of hospitalization, the follow-up stool specimen showed no detection of *E. coli*. Subsequently, the patient’s symptoms improved significantly, and he was discharged on the tenth day of hospitalization.

### 2.2 Antimicrobial susceptibility testing

The minimum inhibitory concentrations (MICs) of antibiotics against *E. coli* were determined using the broth microdilution method. These were interpreted according to the 2025 breakpoints for all agents tested by the Clinical and Laboratory Standards Institute (CLSI), except for aztreonam-avibactam, sitafloxacin, cefoperazone-sulbactam, eravacycline, and tigecycline (Clinical and Laboratory Standards Institute [CLSI], 2025). The MIC of aztreonam-avibactam was interpreted according to the European Committee on Antimicrobial Susceptibility Testing (EUCAST) breakpoints (≤4 mg/L susceptible; ≥4 mg/L resistant) (European Committee on Antimicrobial Susceptibility Testing [EUCAST], 2025). The sitafloxacin MIC was interpreted according to the epidemiological cutoff value (ECOFF) breakpoints (≤0.03 mg/L susceptible; ≥0.06 mg/L resistant) (Zhang et al., 2025). The cefoperazone-sulbactam MIC was interpreted according to the report by Barry and Jones (1988) (≤16 mg/L susceptible, 32 mg/L intermediate, ≥64 mg/L resistant). The MICs of eravacycline and tigecycline were interpreted according to the European Committee on Antimicrobial Susceptibility Testing (EUCAST) breakpoints (≤0.5 mg/L susceptible and >0.5 mg/L resistant, respectively) (European Committee on Antimicrobial Susceptibility Testing [EUCAST], 2025). *E. coli* ATCC 25922 was used as a quality control for antimicrobial susceptibility testing.

### 2.3 Whole-genome sequencing and bioinformatic analysis

Genomic DNA of *E. coli* SE-eco-21 was extracted using a commercial kit (TIANGEN, Beijing, China), according to the manufacturer’s protocols. Bacterial genomic DNA was sequenced using the Illumina MiSeq platform (Illumina Inc.) with paired-end reads (2 × 150 bp) for short-read sequencing, and using the Oxford Nanopore MinION platform (Oxford Nanopore, Oxford, United Kingdom) for long-read sequencing. Sequencing reads were *de novo* assembled using SPAdes software (version 3.13.0) (Bankevich et al., 2012). Antimicrobial resistance genes and plasmid replicon analysis were executed through the Abricate software, referencing the CARD (Zankari et al., 2012) and PlasmidFinder database (Carattoli et al., 2014), respectively. Multilocus sequence typing (MLST) analysis was conducted via the PubMLST database.^1^ Serotyping using ECTyper (version 1.0) with default parameters (Bessonov et al., 2021). Genome annotation was performed using Prokka (Seemann, 2014). The genetic environment structure was analyzed by Easyfig tools (Sullivan et al., 2011).^2^ Comparative genome circle map was conducted by BLAST Ring Image Generator (BRIG) (Alikhan et al., 2011).

### 2.4 Horizontal transfer assays

A plasmid conjugation assay was performed to characterize the *bla*_*NDM–*80_-carrying plasmids. *E. coli* strains EC600 and J53 were used as recipient strains, while *E. coli* SE-eco-21 carrying *bla*_*NDM–*80_ served as the donor strain in the conjugation experiments. Transconjugants were selected on Luria-Bertani (LB) agar supplemented with rifampicin (50 mg/L) and ampicillin (50 mg/L) for *E. coli* EC600, and with sodium azide (50 mg/L) and ampicillin (50 mg/L) for *E. coli* J53. The presence of *bla*_*NDM*_ was confirmed by PCR followed by sequencing. Plasmid DNA was extracted from *E. coli* SE-eco-21 and transferred into *E. coli* DH5α by electroporation. Transformants were selected on Luria–Bertani (LB) agar supplemented with ampicillin (50 mg/L). The presence of *bla*_*NDM*_ was confirmed by PCR and PCR-based sequencing. Antimicrobial susceptibility testing of transformants was determined by the broth microdilution method.

### 2.5 Cloning experiment

Analysis of antibiotic resistance mediated by *bla*_*NDM–*80_ in comparison with *bla*_*NDM–*1_ and *bla*_*NDM–*5_ was investigated by amplifying either the entire open reading frame (primer: F1: 5′-CCATGATTACGAATTCATGGAATTGCCCAATATTATGCACCC-3′; R1: 5′-CGACTCTAGAGGATCCTCAGCGCAGCTTGTCGG-3′) or the complete gene with its native promoter (primer: F2: 5′-TGACCATGATTACGAATTCGGGACTTGTTCGCACCTTCC-3′; R2: 5′-CGACTCTAGAGGATCCTCAGCGCAGCTTGTCGG-3′). Purified PCR amplicons were cloned into PHSG398 vectors and transformed into *E. coli* DH5α. Transformants were selected on LB agar supplemented with ampicillin (50 mg/L). The sequence and phenotype mediated by *bla*_*NDM*_ were verified by PCR-based sequencing and antimicrobial susceptibility test.

### 2.6 Expression and purification of NDM proteins

The sequences of NDM-80, NDM-1, and NDM-5 without the peptide signal region were amplified by PCR using primers *Eco*RI-NDM29-271: 5′-GCAAATGGGTCGCGGATCCGGTGAAAT CCGCCCGACG-3′ and *Bam*HI-NDM29-271: 5′-GTCG ACGGAGCTCGAATTCTCAGCGCAGCTTGTCGG-3′. Purified PCR amplicons were cloned into Pet-28a vectors and transformed into *E. coli* BL21(DE3). NDM Protein was overexpressed in *E. coli* BL21(DE3) overnight at a temperature of 19 °C. After that, the protein was collected and purified by nickel affinity chromatography and imidazole elution. The concentration of NDM protein was determined by measuring the absorbance at 280 nm, and concentrated it to 1.5–2.0 mg/mL.

### 2.7 Steady-state kinetic determinations

Kinetic parameters were determined using purified NDM-80, NDM-1, and NDM-5 in 50 mM HEPES (pH 7.5) supplemented with 100 μM ZnSO4 to detect the hydrolysis of the β-lactams at 25 °C. The real-time absorbances for ceftazidime (257 nm), cefiderocol (259 nm), cefepime (254 nm), imipenem (297 nm), meropenem (298 nm), and aztreonam (318 nm) were detected with a UVProbe spectrophotometer (Kyoto, Japan). The Michaelis–Menten equation was used to calculate and analyze kinetic parameters.

## 3 Results

### 3.1 Antimicrobial susceptibility testing

The results of antimicrobial susceptibility testing revealed that *E. coli* SE-eco-21 exhibited resistance to imipenem, meropenem, meropenem-vaborbactam, ceftolozane-tazobactam, ceftazidime-avibactam, cefepime, ceftazidime, ceftriaxone, ciprofloxacin, cefoperazone-sulbactam, piperacillin-tazobactam, and trimethoprim-sulfamethoxazole. Conversely, the strain demonstrated susceptibility to aztreonam, aztreonam-avibactam, eravacycline, sitafloxacin, tigecycline, colistin and cefiderocol (Table 1).

**TABLE 1:** Minimal inhibitory concentrations (MICs) of clinical strains, transformants, and *E. coli* DH5α receptor bacteria.

Isolates no.	*bla*_NDM_	MIC (mg/L)									
		IPM	MEM	MEV	CZT	CZA	AZA	ERV	AMK	FEP	CAZ
*E. coli* SE-eco-21	*bla*_NDM–80_	8	32	32	>128	>64	0.06	0.25	2	>32	>32
*E. coli* DH5α	–	0.125	<0.03	<0.03	0.5	0.06	<0.03	0.125	2	0.5	0.5
*E. coli* DH5α- SE-plasmidC	*bla*_NDM–80_	8	16	16	>128	>64	0.06	0.125	2	32	>32
*E. coli* DH5α-PHSG398	–	0.125	<0.03	<0.03	0.25	0.25	<0.03	0.125	<1	<0.25	<0.25
*E. coli* DH5α-PHSG398-*bla*_NDM–80_	*bla*_NDM–80_	1	0.125	0.125	>128	32	<0.03	0.125	<1	1	>32
*E. coli* DH5α-PHSG398-*bla*_NDM–5_	*bla*_NDM–5_	1	0.125	0.125	>128	>64	<0.03	0.125	<1	1	>32
*E. coli* DH5α-PHSG398-*bla*_NDM–1_	*bla*_NDM–1_	0.5	0.06	0.125	128	32	<0.03	0.125	<1	0.5	>32
*E. coli* DH5α-PHSG398-promoter-*bla*_NDM–80_	*bla*_NDM–80_	64	64	64	>128	>64	0.06	0.25	2	>32	>32
*E. coli* DH5α-PHSG398- promoter-*bla*_NDM–5_	*bla*_NDM–5_	64	64	64	>128	>64	<0.03	0.125	<1	>32	>32
*E. coli* DH5α-PHSG398- promoter-*bla*_NDM–1_	*bla*_NDM–1_	64	64	64	>128	>64	<0.03	<0.06	<1	>32	>32
**MIC (mg/L)**											
**CRO**			**ATM**	**CIP**	**SIT**	**CSL**	**TZP**	**TGC**	**COL**	**SXT**	**FDC**
>32			<1	>8	4	>128	>256	0.25	0.25	>16	1
<0.25			<1	<0.06	<0.06	<1	4	0.125	0.25	<0.25	<0.03
>32			<1	<0.06	<0.06	>128	>256	0.125	0.25	<0.25	0.125
<0.25			<1	<0.06	<0.06	<1	4	0.125	0.25	<0.25	0.06
16			<1	<0.06	<0.06	8	16	0.125	0.25	<0.25	1
16			<1	<0.06	<0.06	8	32	0.125	0.25	<0.25	1
16			<1	<0.06	<0.06	8	16	0.125	0.25	<0.25	0.5
>32			<1	<0.06	<0.06	>128	>256	0.125	0.25	<0.25	1
>32			<1	<0.06	<0.06	>128	>256	<0.06	0.25	<0.25	1
>32			<1	<0.06	<0.06	>128	>256	0.125	0.25	<0.25	0.5

### 3.2 Whole-genome sequencing assay and characterization of the *bla*_*NDM*_-carrying plasmid

**FIGURE 1:**
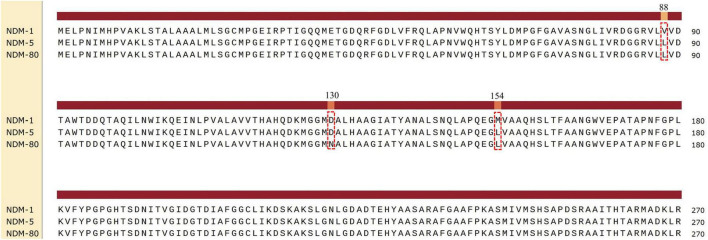
Alignment of NDM-1 (NG_049326.1), NDM-5 (NG_049337.1), and NDM-80 (PV430024.1) amino acid sequences.

**FIGURE 2:**
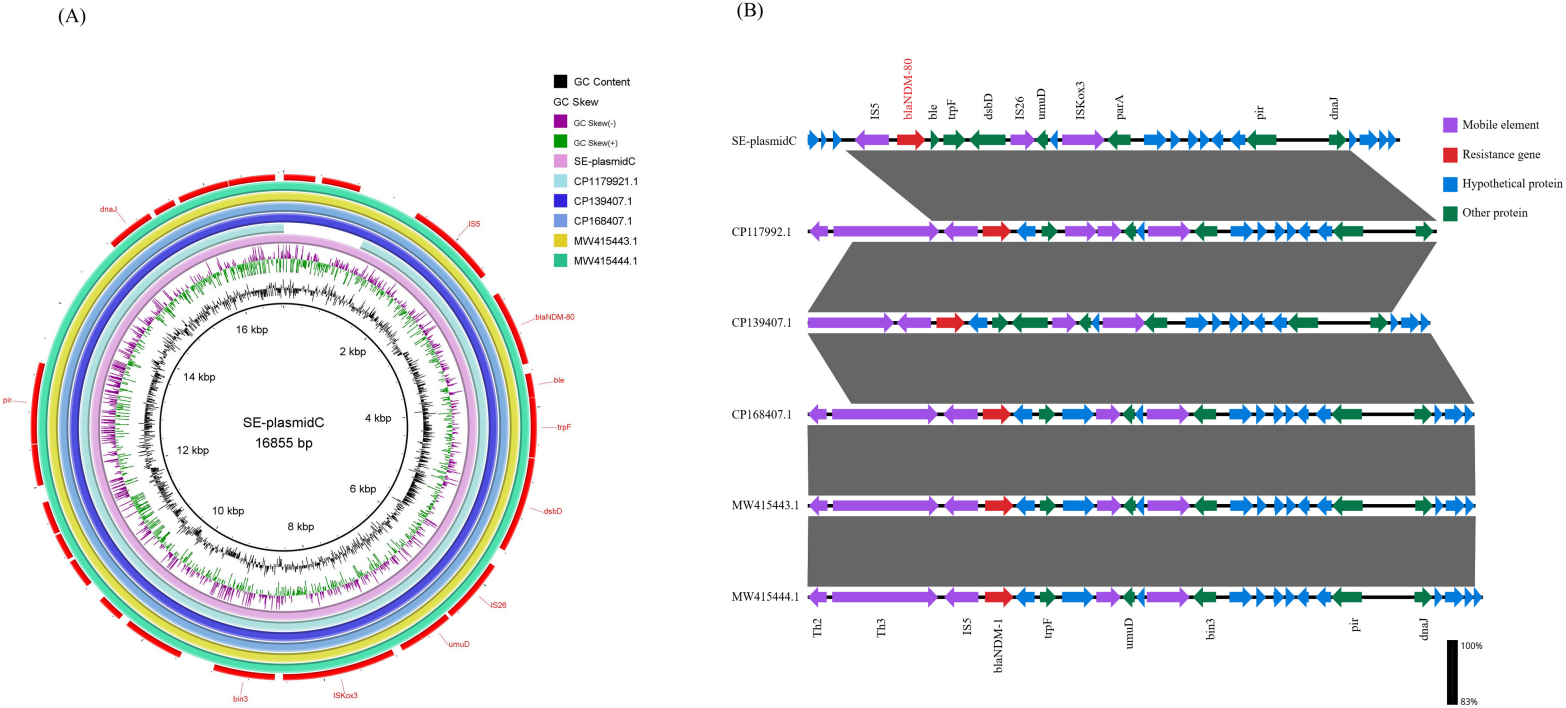
**(A)** Alignments of plasmids. Comparison of the plasmids SE-plasmidC and other IncX3 plasmids using BRIG. **(B)** The genetic environment surrounding *bla*_NDM_ in SE-plasmidC and other IncX3 plasmids.

Next-generation sequencing analysis revealed that *E. coli* SE-Eco-21 belongs to sequence type (ST) 155 and the O4:H51 serotype. One novel NDM-encoding gene, designated *bla*_*NDM–*80_ (Genebank accession: PV430024.1), was identified in *E. coli* SE-eco-21. The *bla*_*NDM–*80_ contains three-point mutations relative to *bla*_*NDM–*1_ (Genebank accession: NG_049326.1) at positions 262 (Gat p 388 (G→A), and 460 (A→C). These mutations generate the amino acid substitutions Val88Leu, Asp130Asn, and Met270Leu. The *bla*_*NDM–*80_ differs from *bla*_*NDM–*5_ (Genebank accession: NG_049337.1) by a single nucleotide at position 388 (G→A), resulting in an amino acid substitution Asp130Asn (Figure 1). Third-generation sequencing revealed that *E. coli* SE-eco-21 harbored a 5,057,113 bp chromosome and four plasmids ranging in size from 4,308 to 184,004 bp (Table 2). The *bla*_*NDM–*80_ was carried by SE-plasmidC, which belongs to the IncX3 plasmid type. The *bla*_*NDM–*80_ was the only antibiotic resistance gene present on SE-plasmidC, which was almost identical (100% query coverage and >99.9% nucleotide identity) to many other IncX3-type plasmids carrying *bla*_*NDM*_, such as plasmids GenBank numbers CP017992.1, CP0139407.1, CP0168407.1, MW415443.1, and MW415444.1 (Figure 2A). The complete genetic structure of *bla*_*NDM–*80_ was *IS*5-*bla*_*NDM–*80_-*ble*-*trpF*-*dsbD*-*IS*26-*nmuD*-*IS*Kox3. Compared to other similar IncX3-type plasmids in the NCBI database, it was found to be absent of the *Tn*2 and *Tn*3 transposons (Figures 2A, B).

**TABLE 2:** Key features of chromosome and plasmids harbored by *E. coli* SE-eco-21.

Sample name	Genome size (bp)	MLST	Serological typing	Plasmid Inc., type	Antibiotic resistance gene(s)
Chromosome	5,057,113 bp	155	O4:H51	NA	*mdf(A)*
SE-plasmidA	184,004 bp	NA	NA	IncHI2, RepA, IncHI2A	*lnu(F)*, *ant(3′′)-Ia*, *qnrS1*, *tet(A)*, *aph(3′′)-Ib*, *aph(6)-Id*, *aph(3′)-Ia*, *aph(4)-Ia*, *aac(3)-IVa*
SE-plasmidB	138,252 bp	NA	NA	IncFIB/IncFIC(FII)	*dfrA14*, *sul3*, *aph(3′)-Ia*, *bla*_TEM–1A_, *sul2*, *aph(3′′)-Ib*, *aph(6)-Id*, *tet(A)*, *floR*
SE-plasmidC	17,067 bp	NA	NA	IncX3	*bla*_NDM–80_
SE-plasmidD	4,308 bp	NA	NA	Col156	NA

### 3.3 Plasmid transformation assay

The results of the plasmid transformation assay (Table 1) show that, compared to recipient *E. coli* DH5α, the MICs of the transformant *E. coli* DH5α- SE-plasmidC (*bla*_*NDM–*80_ positive) to imipenem, meropenem, meropenem-vaborbactam, ceftolozane-tazobactam, ceftazidime-avibactam, cefepime, ceftazidime, ceftriaxone, cefoperazone-sulbactam, and piperacillin-tazobactam increased by between 64 and 1067-fold. This is consistent with the resistance phenotype observed in the original strains. Notably, no *bla*_*NDM–*80_-positive conjugants were detectable when either *E. coli* J53 or *E. coli* EC600 was used as the recipient strain. In this study, the plasmid carrying the *bla*_*NDM–*80_ gene appears to lack the ability to be transmitted by conjugation, probably due to the absence of the type IV secretion system (T4SS), the type IV coupling protein gene (T4CP), and the relaxase.

### 3.4 Functional analysis of *bla*_*NDM–*80_

To compare the antibiotic activity profiles of NDM-80 with those of NDM-5 and NDM-1, the relevant genes were cloned and transformed into *E. coli* DH5α. Two different types of *bla*_*NDM*_-positive transformants were generated (see Table 1). The first type contained only the complete *bla*_*NDM*_ open reading frame and was designated *E. coli* DH5α-PHSG398-*bla*_*NDM–*80_, *E. coli* DH5α-PHSG398-*bla*_*NDM–*5_, and *E. coli* DH5α-PHSG398-*bla*_*NDM–*1_. The second type contained the complete *bla*_*NDM*_ with its native promoter and was designated *E. coli* DH5α-PHSG398-promoter-*bla*_*NDM–*80_, *E. coli* DH5α-PHSG398-promoter-*bla*_*NDM–*5_, and *E. coli* DH5α-PHSG398-promoter-*bla*_*NDM–*1_. The results of the antimicrobial susceptibility test showed that *E. coli* DH5α harboring *bla*_*NDM–*80_, *bla*_*NDM–*5_, or *bla*_*NDM–*1_ exhibited reduced susceptibility to all tested β-lactam antibiotics, including carbapenems, cephalosporins, and β-lactam/β-lactamase inhibitors, when compared with *E. coli* DH5α-PHSG398 (Table 1). Notably, the basal expression of *bla*_*NDM*_ open reading frame under the vector promoter (lacZ) affected the MICs toward imipenem, meropenem, meropenem-vaborbactam, and cefepime in DH5α strains. The *E. coli* DH5α-PHSG398-*bla*_*NDM–*80_ transformant showed similar β-lactam antibiotic resistance to the *E. coli* DH5α-PHSG398-*bla*_*NDM–*5_ transformant. Compared to the *E. coli* DH5α-PHSG398-*bla*_*NDM–*1_ transformant, the *E. coli* DH5α-PHSG398-*bla*_*NDM–*80_ transformant had a twofold increase in the MIC of imipenem, meropenem, cefepime, and cefiderocol. However, these values remain within the susceptible range in the absence of the native promoter. Interestingly, transformants expressing *bla*_*NDM*_ with the native promoter exhibited significantly higher MICs for imipenem, meropenem, meropenem-vaborbactam, and cefepime, suggesting that the wild-type promoter plays a significant role in enhancing resistance to these antibiotics (see Table 1).

### 3.5 Enzyme activity analysis

**FIGURE 3:**
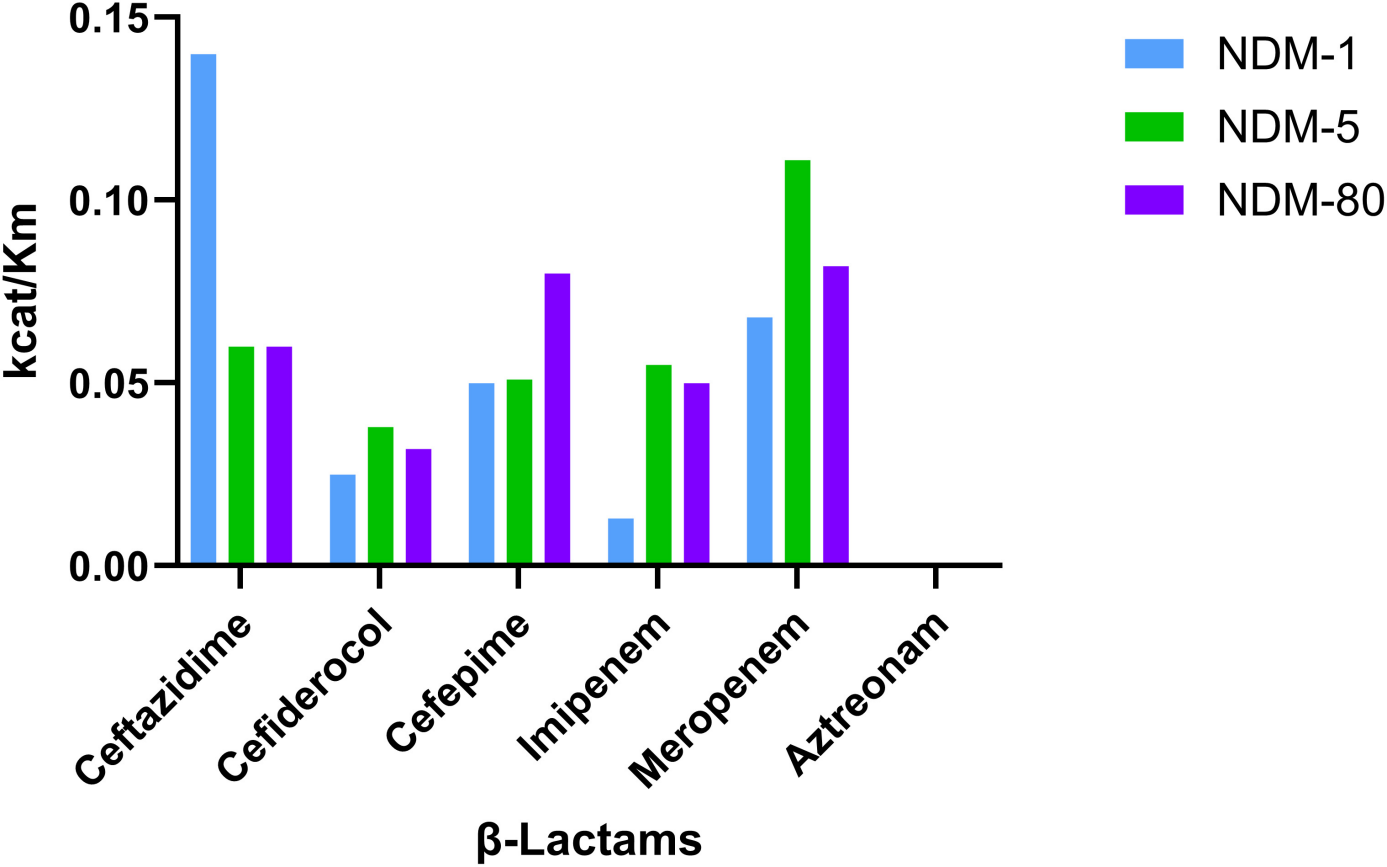
Catalytic efficiency of NDM-1, NDM-5, and NDM-80 against β-lactam antibiotics.

To characterize NDM-80 and investigate the impact of the amino acid point mutation on its enzymatic activity, NDM-80, NDM-5, and NDM-1 were purified, and their kinetic parameters were determined. NDM-80 was found to hydrolyze all of the tested β-lactams except aztreonam (see Figure 3 and Table 3). Compared to NDM-1, NDM-80 exhibited a higher k_(cat)/K_m ratio for cefiderocol, cefepime, imipenem, and meropenem, but a lower k_(cat)/K_m ratio for ceftazidime. Kinetic analysis revealed that NDM-80 probably exhibits higher enzymatic activity toward cefiderocol, cefepime, imipenem, and meropenem, but lower activity toward ceftazidime than NDM-1. Compared to NDM-5, NDM-80 had a lower k_(cat)/K_m ratio for cefiderocol, imipenem, and meropenem, but a higher k_(cat)/K_m ratio for cefepime. This suggests that NDM-80 likely exhibits lower enzymatic activity for cefiderocol, imipenem, and meropenem, but higher enzymatic activity for cefepime than NDM-5.

**TABLE 3:** Steady-state kinetic parameters of purified NDM-1, NDM-5 and NDM-80 enzymes.

β -Lactams	NDM-1			NDM-5			NDM-80		
	**Km (μ M)**	**kcat (s^–1^)**	**kcat/Km (μ M^–1^ s^–1^)**	**Km (μ M)**	**kcat (s^–1^)**	**kcat/Km (μ M^–1^ s^–1^)**	**Km (μ M)**	**kcat (s^–1^)**	**kcat/Km (μ M^–1^ s^–1^)**
Ceftazidime	47.2432	6.509	0.14	177.8154	11.3705	0.06	324.05	20.7	0.06
Cefiderocol	148.414	3.7265	0.025	149.6593	5.6435	0.038	478.9576	15.485	0.032
Cefepime	150.4413	1.51	0.050	153.496	7.8005	0.051	396.1818	32.128	0.08
Imipenem	126.3622	1.6305	0.013	225.5201	14.2675	0.055	698.3814	34.9645	0.050
Meropenem	16.7962	1.1385	0.068	25.4624	2.84	0.111	72.0383	5.9245	0.082
Aztreonam	ND	ND	ND	ND	ND	ND	ND	ND	ND

## 4 Discussion

Carbapenem-resistant Enterobacterales are widespread in the pediatric population, particularly among children in pediatric intensive care units, where they cause infections and pose a serious global public health issue (Fu et al., 2023; Ruvinsky et al., 2022). The production of Class B β-lactamase NDM is the primary mechanism by which CRE isolated from pediatric patients exhibit carbapenem resistance. NDM exhibits significant hydrolytic activity against most β-lactam antibiotics, resulting in limited treatment options for infection control (Han et al., 2020). According to recommendations from the Infectious Diseases Society of America, the preferred treatment options for NDM and other MBL-producing Enterobacterales infections are ceftazidime-avibactam in combination with aztreonam or cefiderocol as monotherapy (Tamma et al., 2024). Aztreonam is resistant to hydrolysis by NDMs, and avibactam is generally effective at inhibiting the activity of other co-produced β-lactamases, such as KPCs, ESBLs, and AmpCs. When used in combination, enhanced antibacterial efficacy can be achieved (Tamma et al., 2024). Cefiderocol, which functions as an iron-chelating cephalosporin, is stable against metalloenzymes and is a significant therapeutic alternative; however, it is not currently available for clinical use in China (Sato and Yamawaki, 2019; Aoki et al., 2018). This study reports a novel *bla*_NDM_ variant, designated *bla*_NDM–80_, identified in carbapenem-resistant *E. coli* for the first time. Antimicrobial susceptibility testing showed that the clinical strain harboring the *bla*_NDM–80_ variant exhibited high-level resistance to all β-lactam antimicrobial agents except for aztreonam, aztreonam-avibactam, and cefiderocol. Furthermore, accumulating evidence indicates that NDM is the only metallo-β-lactamase capable of mediating cefiderocol resistance, primarily via *bla*_NDM_ overexpression, alterations to transferrin, and the emergence of novel mutants (e.g., *bla*_NDM–9_ and *bla*_NDM–35_) (Simner et al., 2022; Nurjadi et al., 2022; Poirel et al., 2022). In our study, molecular cloning results showed that the cefiderocol MIC for the *bla*_NDM–80_-positive transformant (1 mg/L) was double that for the *bla*_NDM–1_-positive transformant (0.5 mg/L), yet both values remain well within the susceptible range. This single dilution increase, which may fall within the inherent variability of the testing method, suggests that the NDM-80 variant probably does not confer a clinically meaningful reduction in cefiderocol susceptibility compared to NDM-1. However, it is crucial to strengthen surveillance of pathogens harboring *bla*_NDM_ variants in order to prevent the emergence of high-level cefiderocol-resistant strains and to inform clinical antimicrobial therapy.

The *bla*_NDM_ resistance genes are typically located on conjugative plasmids, with IncFII and IncX3 plasmids being the most prevalent. This enables them to be disseminated widely among pathogenic bacteria. To date, the NCBI database has documented more than 90 *bla*_NDM_ variants, 49 of which were initially isolated from *E. coli*. Internationally, *bla*_NDM–1_ is the most prevalent variant among *E. coli* isolates, followed by *bla*_NDM–5_, *bla*_NDM–9_, and *bla*_NDM–7_ (Wu et al., 2019). In contrast, *bla*_NDM–5_ is most prevalent in CRE strains isolated in China, followed by *bla*_NDM–1_, with other variants rarely detected (Li et al., 2024; Han et al., 2020). Compared with *bla*_NDM–1_, the *bla*_NDM–80_ variant identified in *E. coli* in this study harbors three amino acid mutations (including V88L, D130N, and M154L). By contrast, only a single amino acid mutation (D130N) is present compared to *bla*_NDM–5_. The *bla*_NDM–80_ is located on an IncX3 plasmid. Previous studies have demonstrated that the IncX3 plasmid can transfer *bla*_NDM_ between different enterobacterial species (Shao et al., 2020; Moussa et al., 2024). Interestingly, no *bla*_NDM–80_-positive conjugants were detectable when *E. coli* J53 or *E. coli* EC600 were used as receptors. Sequence alignment analysis of this plasmid with other IncX3 plasmids carrying *bla*_NDM_ resistance genes revealed that, compared with other plasmids (Figure 2B), the upstream region of *bla*_NDM–80_ lacks the *Tn*2 and *Tn*3 transposons. Accumulating evidence has demonstrated that the *Tn*3 family of transposons plays a crucial role in mediating the horizontal transfer of resistance genes (Tang et al., 2024). Furthermore, the plasmid carrying *bla*_NDM–80_ lacks the T4SS, T4CP, and relaxase. Therefore, we hypothesize that these common factors have rendered the IncX3 plasmid in this study non-conjugative. This may restrict the dissemination of *bla*_NDM–80_ among Enterobacterales.

Different *bla*_NDM_ variants exhibit distinct levels of drug resistance. For example, *bla*_NDM–5_ and *bla*_NDM–7_ enhance resistance to meropenem, imipenem, and ertapenem more than *bla*_NDM–1_ does (Hornsey et al., 2011; Göttig et al., 2013), while *bla*_NDM–9_ and *bla*_NDM–35_ increase resistance to cefiderocol more than *bla*_NDM–1_ does (Gaillot et al., 2023; Poirel et al., 2022). The *bla*_NDM–80_ variant isolated in this study exhibits a similar antimicrobial resistance profile to *bla*_NDM–5_. Studies on crystal structures have shown that the active site of *bla*_NDM_ is located at the bottom of a shallow groove formed by loops L3 and L10 (Tada et al., 2013). In the present study, the other two mutation sites (V88L and D130N), except for the M154L mutation, are not located within this active site. This may account for the similar antimicrobial resistance profiles between *bla*_NDM–80_ and *bla*_NDM–5_. Additionally, electroporation and molecular cloning studies revealed that transformants harboring plasmid vectors with the wild-type promoter upstream of *bla*_NDM_ exhibited significant resistance to imipenem, meropenem, meropenem-vaborbactam, and cefepime. Conversely, transformants lacking this wild-type promoter exhibited only a slight increase in MICs for these antimicrobials, which did not reach the threshold for drug resistance. These findings further confirm that the wild-type promoter upstream of *bla*_NDM_ is a crucial factor in *bla*_*NDM*_-mediated resistance to these agents, consistent with previous research reports (Göttig et al., 2013).

There are several limitations to our study. First, the conjugation experiments were conducted exclusively under controlled *in vitro* conditions and lack validation in clinical settings. The vitro environment differs substantially from the complex physiological milieu *in vivo*. Second, our study lacks therapeutic efficacy testing; antibiotics that are sensitive *in vitro* may not be effective *in vivo*. In conclusion, the present study identified a novel *bla*_NDM_ variant, designated *bla*_NDM–80_, in *E. coli* isolated from a 1-year-old patient. Investigating *bla*_NDM–80_ expands our understanding of *bla*_NDM_ variants, which may arise from the spontaneous adaptive evolution of pathogens under selective antimicrobial pressure, particularly carbapenems. Therefore, establishing a drug resistance surveillance network for pathogens producing NDM enzyme variants is imperative in the future. This will clarify the global incidence, resistance mechanisms, transmission characteristics, and risk factors for infection with these drug-resistant strains, thereby providing a scientific basis for curbing the further dissemination of such resistant pathogens.

## Data Availability

The sequence data has been uploaded to the NCBI Genbank public database. GenBank accession number for blaNDM-80 is PV430024.1 and GenBank accession number for SE-plasmidC is PX608136.

## Footnotes

1
https://pubmlst.org

2
http://mjsull.github.io/Easyfig
